# Discovery of *Neonrosellavitiata* (Darwin) and *Newmanellaspinosus* Chan & Cheang (Balanomorpha, Tetraclitidae) from the Andaman Sea, eastern Indian Ocean

**DOI:** 10.3897/zookeys.833.30689

**Published:** 2019-03-25

**Authors:** Woranop Sukparangsi, Ashitapol Pochai, Chinnakit Wongkunanusorn, Salinee Khachonpisitsak

**Affiliations:** 1 Department of Biology, Faculty of Science, Burapha University, 169 Long-Hard Bangsaen Road, Saen Suk, Mueang, Chon Buri, 20131 Thailand; 2 Takuapa Senanukul School, 15 Phet Kasem Road, Bang Nai Si, Takua Pa, Phang-Nga, 82110 Thailand; 3 Biology Program, Department of Biology, Faculty of Science, Burapha University, 169 Long-Hard Bangsaen Road, Saen Suk, Mueang, Chon Buri, 20131 Thailand

**Keywords:** Acorn barnacle, Cirripedia, Crustacea, Newmanellinae, Sessilia

## Abstract

In this present study, distantly related acorn barnacle species in the subfamily Newmanellinae (Cirripedia, Thoracica, Tetraclitidae), including *Neonrosellavitiata* (Darwin, 1854) and *Newmanellaspinosus* Chan & Cheang, 2016, were discovered in the Andaman Sea of Thailand. *Neo.vitiata* can be readily distinguished from other newmanellids by shell plate and operculum morphology (external shell, tergum geometry, and pattern of parietal tube) and arthropodal characters (presence of basi-dorsal point at base of penis and triangular spines on cirri, setal type, and mouth parts). Both species were found to share overlapping territories on rocks at the rockweed zone, an area submerged under seawater most of the time throughout the year. This study highlights the first discovery of *Neonrosella* in the eastern Indian Ocean, whose ultrastructure compared to *Newmanella* is redescribed and illustrated here based on scanning electron microscopy.

## Introduction

The genus *Neonrosella* Jones, 2010 contains only one species, *Neonrosellavitiata* (Darwin, 1854). This species was placed and repositioned in different taxa of family Tetraclitidae. Originally, it was described in the genus *Tetraclita* Schumacher, 1817 belonging to subfamily Tetraclitinae Gruvel, 1903, as *Tetraclitavitiata* by Darwin (1854). For more details of description of *T.vitiata*, see Rosell (1972). Ikeya and Yamaguchi (1993) then placed *T.vitiata* alongside with *T.coerulescens* (Spengler, 1970) into the genus *Newmanella* Ross, 1969 (Ikeya and Yamaguchi 1993).

Later, a revision of species of the superfamily Tetraclitoidea Gruvel, 1903 was done by Ross and Perreault (1999). Based on the difference in shell morphology compared with species of the genus *Newmanella*, they moved *Newmanellavitiata* (Darwin, 1854) to a newly proposed genus *Yamaguchiella* Ross & Perreault, 1999, and established this barnacle as a new subgenus Yamaguchiella (Rosella) Ross & Perreault, 1999. Thus, this species was renamed as Yamaguchiella (Rosella) vitiata (Darwin, 1854). In addition, they also placed both genera *Newmanella* and *Yamaguchiella* in a newly proposed subfamily Newmanellinae Ross & Perreault, 1999.

Afterwards, Jones (2010) proposed *Neonrosella* Jones, 2010 to replace *Rosella*, as that name was already assigned to a genus of curculionid beetles (Insecta, Coleoptera) by Whitehead (1977; in Clark et al. 1977). Thus, Jones (2010) renamed this species as Yamaguchiella (Neonrosella) vitiata (Darwin, 1854).

Recently, the subgenusNeonrosella was elevated to generic level by Chan and Cheang (2016) based on a phylogenetic analysis to clearly separate Yamaguchiella (Yamaguchiella), which is closer related to *Tetraclitasingaporensis* Chan, Tsang & Chu, 2007, from Yamaguchiella (Neonrosella), which is closer related to *Tetraclitaehsani* Shahdadi, Chan & Sari, 2011 (Tsang et al. 2015). The subspecies Yamaguchiella (Neonrosella) vitiata was thus elevated to species status as *Neonrosellavitiata* (Chan and Cheang 2016).

The genus *Newmanella* was established by Ross (1969) for a group of low intertidal to subtidal tetraclitid barnacles with *Balanusradiata* Bruguière, 1789 as the type species (Ross 1969: 242), later known as *Newmanellaradiata* (Bruguière, 1789) and recently redescribed by Chan and Cheang (2016). Ross and Perreault (1999) proposed the classification for the subfamily Newmanellinae and placed *Newmanella* into that subfamily as well as described a new species *Newmanellakolosvaryi* Ross & Perreault, 1999 from the east coast of Panama in the western Atlantic. Recently, *Newmanellaspinosus* was described as a new species from the western Pacific (Taiwan) by Chan and Cheang (2016). Hence, the genus *Newmanella* is currently represented by four species: *New.hentscheli* Kolosvary, 1942, *New.kolosvaryi*, Ross & Perreault, 1999, and *New.radiata* (Bruguière, 1789) from the Atlantic waters of South America (Bruguière 1789; Kolosvary 1942; Ross and Perreault 1999) and *New.spinosus* Chan & Cheang, 2016 from the western Pacific and the Andaman Sea, eastern Indian Ocean (Chan and Cheang 2016; Pochai et al. 2017, respectively).

A recent examination of acorn barnacle specimens from the Andaman Sea, southern Thailand, recognized two morphologically similar newmanellin species from the same area of the Na-Tai rocky shore (Phang-Nga Province); *Neonrosellavitiata* is new to Thailand and *Newmanellaspinosus* is found next to *Neo.vitiata* at lowest low tide point. Both species are redescribed herein, based on shell plate morphology and arthropodal characters using scanning electron microscopy (SEM). This is also the first illustration of *Neo.vitiata* in its ultrastructure, providing clear observation of this barnacle for taxonomic identification. Both species are compared with the detailed redescription of *New.radiata* provided by Chan and Cheang (2016).

## Materials and methods

This study is based upon material collected from the Andaman Sea at Na-Tai rocky shore, Phang-Nga Province, southern Thailand, in March 2018. Samples were collected by hand picking and were transferred into plastic containers containing 95% ethanol. In the laboratory, specimens were transferred into clean 95% ethanol for storage. Specimens were examined under a compound microscope and stereomicroscope and later selected for dissection. All taxonomically important characters, shell plate morphology, and arthropodal characters were dissected and investigated with LEO 1450 VP scanning electron microscope on gold-coated specimens at Microscopic Center, Faculty of Science, Burapha University.

Specimens are preserved in 95% ethanol and have been deposited in the Zoological Collections of Burapha University, Thailand (ZCBUU).

The general terminology of the shell morphology and arthropodal characters follows Ross (1969), Rosell (1972), Ross and Perreault (1999), and Chan and Cheang (2016). The final images were processed with Adobe Photoshop CS6 and Adobe Illustrator CS6. Abbreviations used to denote shell morphology and arthropodal characters are explained directly in figure captions.

### Museum and collection acronyms

**NMNS** National Museum of Natural Science, Taichung, Taiwan

**ZCBUU** Zoological Collections of Burapha University, Thailand

## Taxonomy

### Order Sessila Lamarck, 1818

#### Suborder Balanomorpha Pilsbry, 1916

##### Superfamily Tetraclitoidea Gruvel, 1903

###### Family Tetraclitidae Gruvel, 1903

####### Subfamily Newmanellinae Ross & Perreault, 1999

######## Genus Neonrosella

Taxon classificationAnimaliaSessiliaTetraclitidae

Jones, 2010, monotypic

######### Type species.

*Neonrosellavitiata* (Darwin, 1854)

######## Redescription of Neonrosella vitiata

Taxon classificationAnimaliaSessiliaTetraclitidae

(Darwin, 1854)

Figs 1 , 2 , 3 , 4

Tetraclita vitiata Darwin, 1854: 340–341, Pl. 11, fig. 3a-e; Hoek 1913: 256; Broch 1922: 339–341, text fig. 73a-c; Hiro 1936: 635; 1937: 67, text fig. 13a & d.
Tetraclita (Tetraclita) vitiata : Rosell 1972: 214.
Newmanella vitiata (Darwin, 1854) Yamaguchi, in Ikeya and Yamaguchi 1993: 93; Jones et al. 1990: 14.
Yamaguchiella (Rosella) vitiata (Darwin, 1854): Ross and Perreault 1999: 5.
Yamaguchiella (Neonrosella) vitiata (Darwin, 1854): Jones 2010: 14.

######### Material examined.

13 specimens, southern Thailand, Andaman Sea in the eastern Indian Ocean, Phang-Nga Province, Na-Tai District, Na-Tai rocky shore, 20 Mar 2018, A Pochai leg. ZCBUU-CP-024-036.

######### Diagnosis.

Parietes white with dark orange spots or longitudinal stripes. Tergum with broad spur. Area with lateral tergal depressor crests on basal margin long and carrying numerous and deep crests. Lateral scutal depressor crests numerous and deep. Cirrus II with equal rami. Cirrus III antenniform in both rami; lesser curvature with hook-like spines. Lesser curvature of cirrus IV without spines. Maxillule with two large spines and two smaller spines before notch; five pairs of spines and a cluster of 12 spines after notch. Mandible with five teeth; the third teeth tridentate; the fourth teeth quadridentate and the fifth teeth close to the fourth teeth; seven smaller setae on lower margin; without setae under inferior angle. Labrum with three canine-like teeth on each cutting margin. Penis with basi-dorsal point.

######### Description.

**Figure 1.**
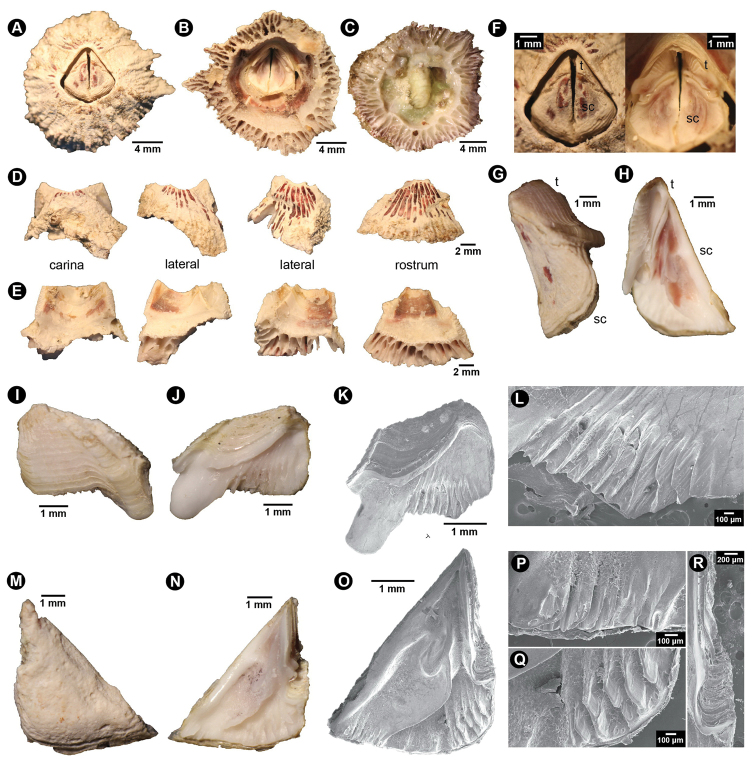
Shell plate and operculum morphology of *Neonrosellavitiata* (Darwin, 1854) **A** anterior view of shell **B** basal view of shell without body tissue **C** basal view of shell with body tissue intact **D** external view of individual shell plates (parietes) **E** internal view of individual shell plates **F** (left) close-up on external view of operculum (right) internal view of intact operculum **G** external view of operculum (one side of both tergum and scutum) **H** internal view of operculum (one side of both tergum and scutum) **I–L** tergum **I** external view **J** internal view **K**SEM of internal view **L** close-up on crests for lateral tergal depressor muscle M)–R) scutum **M** external view **N** internal view **O**SEM of internal view **P** close-up of crests at edge of basal margin (bm) close to occludent margin (om) **Q** close-up of crests for lateral scutal depressor muscle **R** close-up of articular ridge (ar).

Peduncle absent. Body length 2–3 cm. Shell white with orange longitudinal lines; low conic; composed of four shell plates including one carina, two laterals, and one rostrum (Fig. 1A, D, E). Base calcareous with parietal tubes; two rows of irregular shape and size of parietal tubes; inner laminar compartment carrying larger parietal tubes that its intraparietal septum radiating to the outer laminar; outer laminar compartment carrying three smaller and horizontal parietal tubes between larger tubes from inner laminar (Fig. 1B, C). External shell plate ornamented with rough and white with orange longitudinal striation; some exhibited decolouration or erosion of shell plate but carina always possesses 4–5 remnants of orange spots close to the orifice (Fig. 1D). Basal margin of each shell plate irregularly undulated. Internal shell plate smooth and white; interior part close to orifice oranges and with horizontal striation (Fig. 1E). Orifice kite-shaped or pentagonal (Fig. 1F). External surface of opercular plates white with irregular orange-brown spots (Fig. 1F, G). Internal surface of opercular plates mostly white with orange (Fig. 1F, H). Tergum smaller than scutum (Fig. 1G, H). Tergum triangular to polygonal shaped; dorsal surface with horizontal lines; longitudinal furrow on dorsal side broad. Spur of tergum broad with rounded tip. Scutal margin smooth without teeth. Basal margin of tergum longer than carina margin or area with lateral depressor muscle crests thick (ten crests). Tergal articular ridge with broad width but low ridge and thus when articulated, tergum occupies small area of scutum (Fig. 1I–L). Scutum triangular with height similar to width. External surface of scutum without horizontal striation. Ventral surface of scutum with long adductor ridge. Lateral scutal depressor crests deep and numerous (five crests) (Fig. 1M–R).

**Figure 2.**
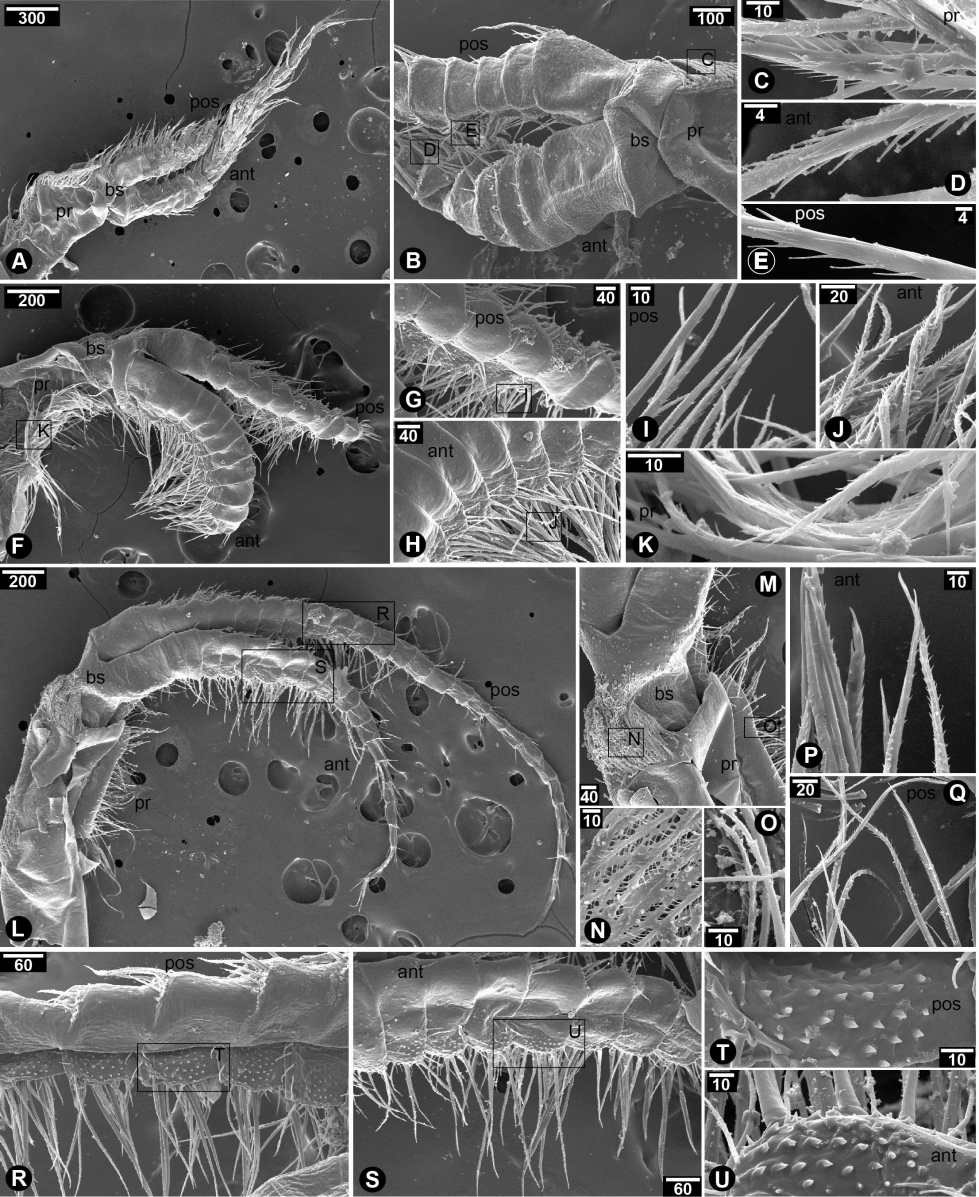
SEM showing cirral morphology of *Neonrosellavitiata* (Darwin, 1854) **A–E** cirrus I **A** overview of cirrus I morphology **B** close-up at proximal region of cirrus I **C** serrulated seta on posterior side of protopod **D** serrulated seta on anterior ramus **E** serrulated seta on posterior ramus **F–K** cirrus II **F** overview of cirrus II morphology **G** posterior ramus **H** anterior ramus **I**serrulated seta on posterior ramus **J** serrulated setae on anterior ramus **K** serrulated seta on anterior side of protopod **L–U** cirrus III **L** overview of cirrus III morphology **M** close-up on basipod and protopod **N** plumose seta on posterior side of basipod **O** plumose seta on anterior side of protopod **P** anterior seta on anterior ramus **Q** posterior seta on posterior ramus **R** posterior ramus **S** anterior ramus **T** close-up on posterior ramus showing spines on lesser curvature **U** close-up on anterior ramus showing spines on lesser curvature. Abbreviations: pr, protopod; bs, basipod; pos, posterior ramus; ant, anterior ramus. Scale bars in µm.

**Figure 3.**
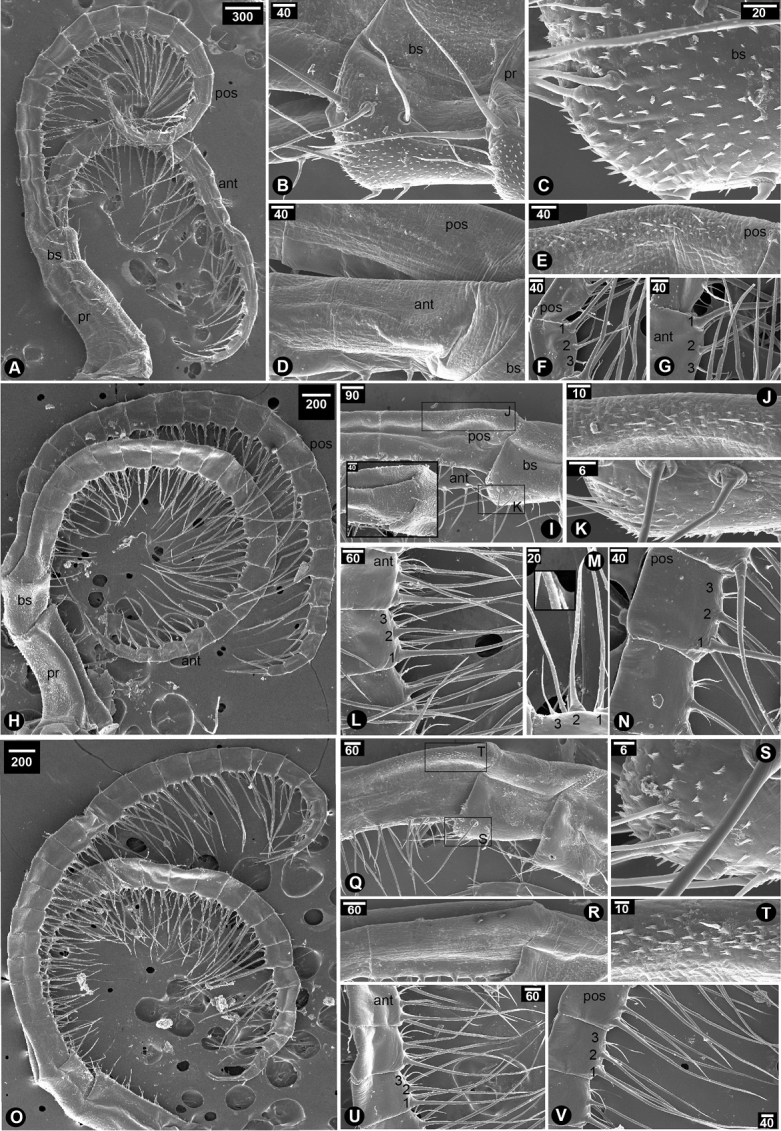
SEM showing cirral morphology of *Neonrosellavitiata* (Darwin, 1854) **A–G** cirrus IV **A** overview of cirrus IV morphology **B** close-up at basis and protopod **C** basipod with spines **D** greater curvature of both rami **E** close-up on spines on posterior ramus **F** lesser curvature of posterior ramus (1, 2 and 3 indicating first pair of long serrulated setae-longest, second pair of serrulated setae-second longest and third pair of simple setae-shortest, respectively) **G** lesser curvature of anterior ramus (number described as F) **H–N** cirrus V **H** overview of cirrus V morphology **I** proximal region showing greater curvature of both rami **J** close-up on greater curvature of posterior ramus carrying spines **K** basipod with spines **L** lesser curvature of anterior ramus **M** close-up on a segment on lesser curvature of anterior ramus showing type of setae (inset showing serrulated setae and number as described in F) **N** lesser curvature of posterior ramus (number as described in F) **O–V** cirrus VI **O** overview of cirrus VI morphology **Q** greater curvature of posterior ramus **R** greater curvature of both rami **S** basipod with spines **T** close-up on spines on posterior ramus **U** lesser curvature of anterior ramus **V** lesser curvature of posterior ramus (number described as F). Abbreviations: pr, protopod; bs, basipod; pos, posterior ramus; ant, anterior ramus. Scale bars in µm.

Cirrus I with unequal rami; anterior ramus (20-segmented) length longer than that of posterior ramus (10-segmented), approximately 2.5 times (Fig. 2A); intermediate segments of posterior ramus normal or not protuberant; greater and lesser curvature of both rami without spine; basipod without spines; protopod with serrulated setae on the posterior side; serrulated setae found in both rami (Fig. 2B–E). Cirrus II with equal rami and similar length (both rami with 10-segmented) (Fig. 2F); lesser curvature of both rami without spines (Fig. 2G–H); posterior ramus with serrulated setae (Fig. 2I); anterior ramus with serrulated setae along the entire length from apex to basipod and bi-pinnate setae on distal segments near apex (Fig. 2J); protopod with long serrulated setae on anterior side (Fig. 2K). Cirrus III with unequal rami; posterior ramus (27-segmented) longer than anterior ramus (19-segmented) about 1.5 fold; both rami antenniform (Fig. 2L); basis without spine (Fig. 2M); basis with plumose setae on posterior side (Fig. 2N); protopod with plumose setae on anterior side (Fig. 2O); serrulated and bidentate setae found in both rami (Fig. 2P, Q); lesser curvature of proximal region of both rami carrying spines (7-segmented on anterior ramus and 4-segmented on posterior ramus) (Fig. 2R, S); spines on both rami with hook-like shaped and thick (Fig. 2T, U). Cirrus IV–VI with equal and long rami (Fig. 3A, H, O); basis of cirrus IV–VI carrying triangular and slender spines (Fig. 3B, C, K, S); only first proximal segment of greater curvature of posterior ramus carrying triangular and slender spines (Fig. 3E, J, T). Cirrus IV, anterior ramus 17-segmented, posterior ramus 18-segmented (Fig. 3A). Cirrus V–VI, anterior ramus 22-segmented, posterior ramus 22-segmented (Fig. 3H, O). Lesser curvature of Cirrus IV–VI without spines and carrying two pairs of long serrulated setae and one pair of shorter simple setae (Fig. 3F, G, L, M, N, U, V).

**Figure 4.**
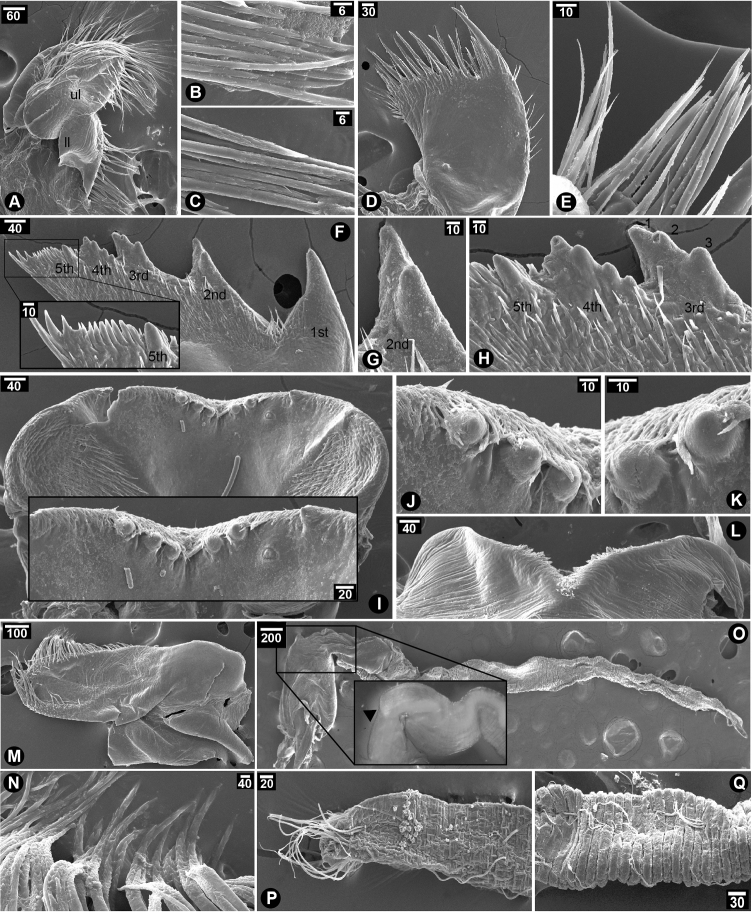
SEM showing mouth parts of *Neonrosellavitiata* (Darwin, 1854) **A–C** maxilla **A** overview of maxilla morphology **B** serrulated setae on upper lobe **C** serrulated setae on lower lobe **D–E** maxillule **D** overview **E** spines and serrulated setae on inferior angle **F–H** mandible **F** overview of mandible morphology and inset showing close-up of lower margin **G** bidentate 2^nd^ teeth **H** tridentate 3^rd^ teeth, quadri-dentate 4^th^ teeth and single 5^th^ teeth **I–L** labrum **I** overview of labrum morphology of interior labrum and inset showing close-up of teeth on labrum **J** close-up of teeth on right side of labrum **K** close-up on teeth on left side of labrum **L** exterior side of labrum **M–N** mandibular palp **M** overview of mandibular palp morphology **N** serrulated setae on superior margin **O–Q** penis **O** overview of whole penis on side view and inset showing basi-dorsal point (arrow head) on base of the penis **P** close-up on apex of penis **Q** annulation along penis. Abbreviations: ul, upper lobe of maxilla; ll, lower lobe of maxilla. Scale bars in µm.

Maxilla bi-lobate; upper lobe covered with densely packed serrulated setae; lower lobe with a few serrulated setae carrying more packed setules (Fig. 4A–C). Maxillule with U-shaped notch; two large spines and two small spines before notch; five pairs of small and slender spines after notch (Fig. 4D); cutting edge after notch carrying another 12 smaller spines followed by a cluster of serrulated setae (Fig. 4E). Mandible with five teeth; the first teeth largest; the second teeth bidentate; the third teeth tridentate; the fourth teeth quadridentate; the fifth teeth only single close to the fourth teeth; lower margin narrow with a pack of seven small setae followed by three larger setae close to inferior angle; no setae under inferior angle; simple setae scattered on surface of mandible (Fig. 4F–H). Labrum with V-shaped notch; three canine-shaped teeth with densely packed simple setae on each side of cutting margin (Fig. 4I–L). Mandibular palp rectangular with serrulated setae on superior margin (Fig. 4M, N). Penis long with annulation with basi-dorsal point on the dorsal side of penis base (Fig. 4O inset); a few simple setae scattered randomly along whole length; two bundles of simple and long setae found at the tip of penis (Fig. 4O–Q).

######### Habitat.

*Neonrosellavitiata* was collected only during the lowest tide (March) of the year and at the lowest littoral zone, an area submerged most of the time throughout the year. It was found on rocks covered with seaweed, densely packed green and red algae, hydroids, sponges, limpets, other acorn barnacles including *Tetraclita* species at the algal crust zone of the intertidal region. The barnacles were found mostly in solitary form, in connection with *Newmanellaspinosus*, or with conspecifics as small colonies of only two or three individuals per colony.

######### Distribution.

Great Barrier Reef (Raine’s Islet), Australia (Darwin 1854); Lucipara Islands, Banda Sea (Hoek 1913), Zamboanga, Philippines (Broch 1922); Goram Island (Hiro 1936); Oropusyakaru and Madarai Islands (Hiro 1937); Philippines (Rosell 1972); Singapore (Jones and Hosie 2016) and Andaman Sea of eastern Indian Ocean, Phang-Nga Province, southern Thailand (new record).

######## Genus Newmanella

Taxon classificationAnimaliaSessiliaTetraclitidae

Ross, 1969

######### Type species.

*Newmanellaradiata* (Bruguière, 1789). Additional species: *New.hentscheli* Kolosvary, 1942, *New.kolosvaryi* Ross & Perreault, 1999, *New.spinosus* Chan & Cheang, 2016.

######## Redescription of Newmanella spinosus

Taxon classificationAnimaliaSessiliaTetraclitidae

Chan & Cheang, 2016

Figs 5 , 6 , 7 , 8

Newmanella radiata . Chan et al. 2009: 199, fig. 170; Shuto and Hayashi 2013: 159, fig. 3c (non New.radiata (Bruguière 1789).
Newmanella sp. Tsang et al. 2015: 325, fig. 1A, 327 fig. 2.
Newmanella spinosus Chan & Cheang, 2016: 212–220, figs 9–15.

######### Type.

NMNS-006535-00001, deposited in NMNS (not examined).

######### Material examined.

17 specimens, southern Thailand, Andaman Sea in the eastern Indian Ocean, Phang-Nga Province, Na-Tai District, Na-Tai rocky shore, 20 Mar 2018, A Pochai leg. ZCBUU-CP-007-023.

######### Diagnosis.

Parietes and opercular plates green on external and internal surfaces. External shell plate with numerous radiating or longitudinal lines extending from apex to base. Scutal margin of tergum with serrated teeth and broad spur with cutting edges. Cirrus II with equal rami and slight curvature of both rami carrying triangular spines. Basis of cirri IV–VI without spines. Greater curvature of both anterior ramus and posterior ramus of Cirrus IV with triangular spines. Mandible with five teeth, the third teeth bidentate, the fourth teeth with serrations and small teeth along the edge, and the fifth teeth sits on the middle of lower margin surrounded by other small spines. Labrum with four teeth on each cutting margin. Penis without basi-dorsal point.

######### Description.

**Figure 5.**
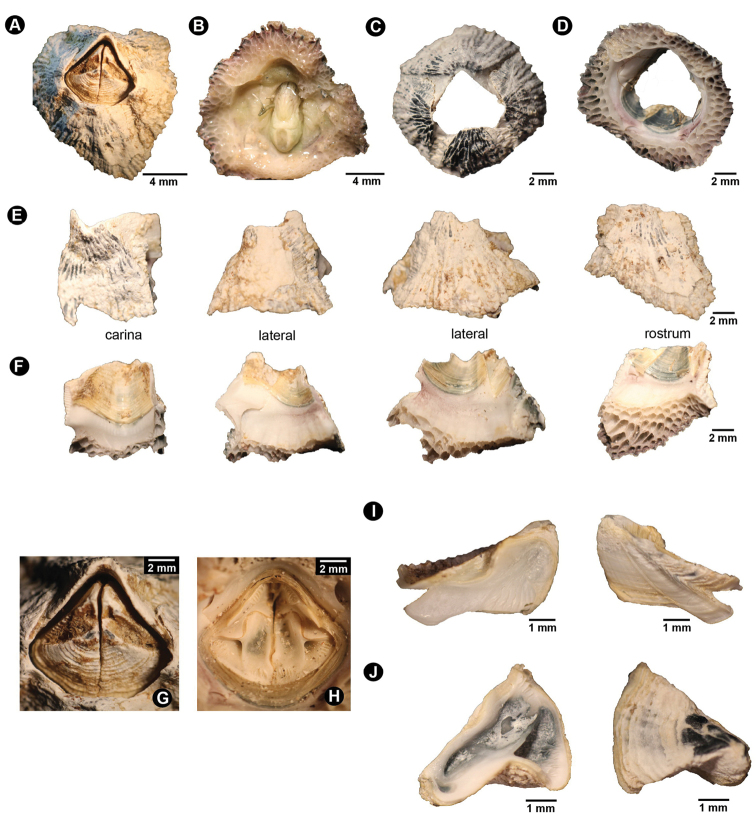
Shell plate and operculum morphology of *Newmanellaspinosus* Chan & Cheang, 2016 **A** anterior view of shell **B** basal view of shell with body tissue **C** external and anterior view of shell without body tissue **D** internal and basal view of shell plates showing parietal tubes **E** external view of parietes **F** internal view of parietes **G** close-up on orifice and exterior opercular plates **H** internal view of operculum without intact tissues **I** internal view (left) and external view (right) of tergum **J** internal view (left) and external view (right) of scutum.

Peduncle absent. Body length 2–3 cm. Shell green with longitudinal folds or lines from orifice toward base or radiating lines; low conic; composed of four shell plates including one carina, two laterals and one rostrum. Base calcareous with parietal tubes; three rows of irregular shape and size of parietal tubes (Fig. 5A–F). External shell plate with longitudinal fold or striation from apex to base; some exhibited decolouration or erosion of shell plate. Basal margin of each shell plate irregularly undulated (Fig. 5E). Internal shell plate smooth and white to pale green; interior part close to orifice green and with some white horizontal striations (Fig. 5F). Orifice pentagonal (Fig. 5G). External surface of opercular plates white with irregular green spots or lines (Fig. 5G). Internal surface of opercular plates mostly white with green, in particular scutum (Fig. 5H). Tergum smaller than scutum (Fig. 5I, J). Tergum triangular with clear spur protruding from basal margin; dorsal surface with horizontal lines; longitudinal furrow on dorsal side broad connected to spur. Spur of tergum broad with cutting edge tip. Scutal margin with serrated teeth. Basal margin of tergum with lateral depressor muscle crests thick (9–10 crests); tergal articular ridge with narrow width (Fig. 5I). Scutum triangular with height 1.3 times base. External surface of scutum with horizontal lines. Lateral depressor crest deep and numerous (5–8 crests) (Fig. 5J).

**Figure 6.**
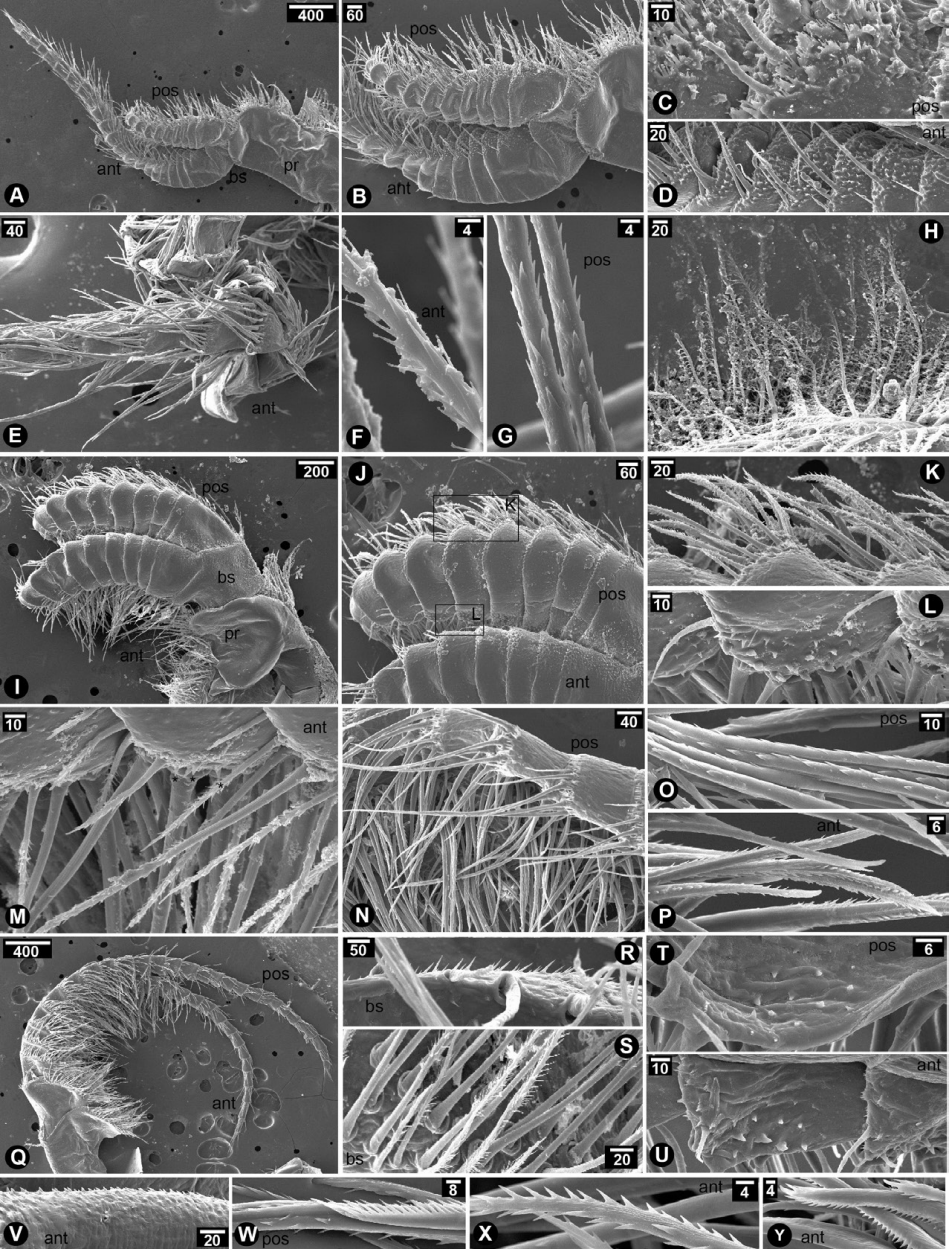
SEM showing cirral morphology of *Newmanellaspinosus* Chan & Cheang, 2016 **A–H** cirrus I **A** overview of cirrus I morphology **B** close-up at proximal region of cirrus I **C** close-up on posterior ramus **D** close-up on anterior ramus **E** apex of anterior ramus **F** serrulated setae on anterior ramus **G** serrulated setae on posterior ramus **H** plumose setae on posterior side of protopod **I–P** cirrus II **I** overview of cirrus II morphology **J** proximal region of cirrus II **K** serrulated and bidentate setae on greater curvature of posterior ramus **L** hook-like triangular spines on lesser curvature of posterior ramus **M** spines (asterisks) on lesser curvature of anterior ramus **N** close-up on apex on posterior ramus **O** serrulated setae on posterior ramus **P** serrulated and bidentate setae on anterior ramus **Q–Y** cirrus III **Q** overview of cirrus III morphology **R** slender spines on basipod **S** plumose setae on anterior side of basipod **T** hook-like triangular spines on lesser curvature of posterior ramus **U** hook-like triangular spines on lesser curvature of anterior ramus **V** greater curvature of anterior ramus with spines **W** serrulated setae and bidentate setae on posterior ramus **X** serrulated setae on anterior ramus **Y** bidentate setae on anterior ramus. Abbreviations: pr, protopod; bs, basipod; pos, posterior ramus; ant, anterior ramus. Scale bars in µm.

**Figure 7.**
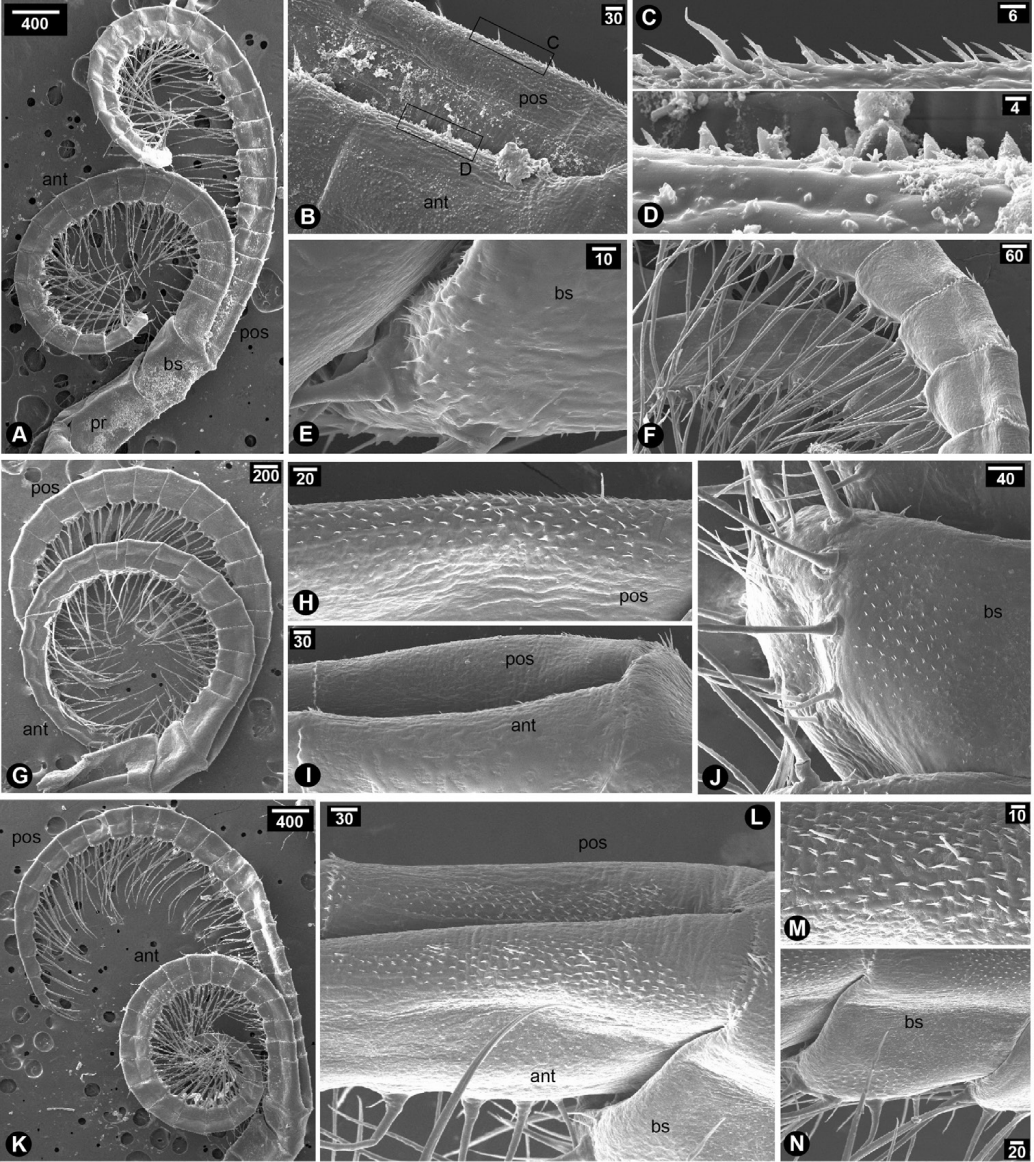
SEM showing cirral morphology of *Newmanellaspinosus* Chan & Cheang, 2016 **A–F** cirrus IV **A** overview of cirrus IV morphology **B** close-up on proximal region of greater curvature **C** slender spines on greater curvature of posterior ramus **D** triangular spines on greater curvature of anterior ramus **E** close-up on basipod showing denticles without spine **F** lesser curvature of both rami showing two pairs of long serrulated setae and a pair of simple setae **G–J** cirrus V **G** overview of cirrus V morphology **H** greater curvature of posterior ramus showing spines **I** greater curvature of both rami (the other side) without spine **J** close-up on basipod without spine (only denticles) **K–N** Cirrus VI **K** overview of cirrus VI morphology **L** greater curvature of both rami **M** close-up on slender spines on greater curvature of anterior ramus **N** close-up on basipod without spine. Abbreviations: pr, protopod; bs, basipod; pos, posterior ramus; ant, anterior ramus. Scale bars in µm.

Cirrus I with unequal rami; anterior ramus (21-segmented) length approx. twice as long as posterior ramus (10-segmented) (Fig. 6A); intermediate segments of posterior ramus normal or not protuberant (Fig. 6B); greater and lesser curvature of both rami without spines (Fig. 6C, D); basipod without spines; serrulated setae found in both anterior and posterior rami (Fig. 6F, G); protopod on the posterior side with plumose setae (Fig. 6H). Cirrus II with equal rami and similar length (both rami with 10-segmented) (Fig. 6I); greater curvature of posterior ramus with serrulated setae (Fig. 6J, K); lesser curvature of posterior ramus with hook-like triangular spines (Fig. 6L) and lesser curvature of anterior ramus with slender spines and serrulated setae (Fig. 6M); apex of posterior ramus with long serrulated setae (Fig. 6N, O); apex of anterior ramus with bi-pinnate setae (Fig. 6P). Cirrus III with unequal rami; posterior ramus (26-segments) longer than anterior ramus (22-segmented, approximately 1.2 times; both rami antenniform (Fig. 6Q); basipod with spines (Fig. 6R) and anterior side of basipod with serrulated setae (Fig. 6S); weak curvature of both rami with hook-like triangular spines (Fig. 6T, U); greater curvature of anterior ramus with short spines (Fig. 6V); Both rami with serrulated setae and bidentate setae (Fig. 6W–Y). Cirrus IV–VI with semi-equal and long rami (Fig. 7A, G, K); basis of cirrus IV–VI without spines, only denticles observed (Fig. 7E, J, N). Cirrus IV, anterior ramus 20-segmented posterior ramus 21-segmented (Fig. 7A); Greater curvature of posterior ramus with slender spines (Fig. 7B, C); Greater curvature of anterior ramus with broad triangular spines (Fig. 7B, D); each segment carries two pairs of long serrulated setae and one pair of shorter simple setae (Fig. 7F). No spine on each segment at lesser curvature side. Cirrus V, anterior ramus 19-segmented posterior ramus 20-segmented (Fig. 7G); Greater curvature of posterior ramus with slender spines (Fig. 7H) while no spines on anterior ramus (Fig. 7I). Cirrus VI, anterior ramus 24-segmented, posterior ramus 26-segmented (Fig. 7K); greater curvature of both rami with slender spines (Fig. 7L, M).

**Figure 8.**
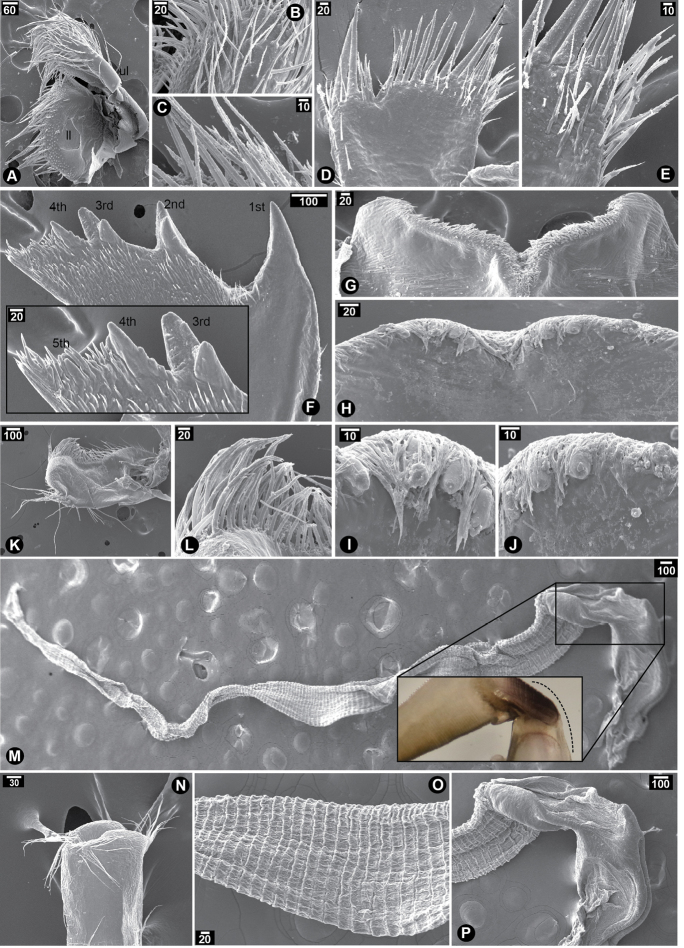
SEM showing mouth parts of *Newmanellaspinosus* Chan & Cheang, 2016 **A–C** maxilla **A** overview of maxilla morphology showing upper lobe (ul) and lower lobe (ll) **B** serrulated setae on upper lobe **C** serrulated setae on lower lobe **D–E** maxillule **D** overview of maxillule morphology **E** serrulated setae on inferior angle of maxillule **F** mandible (inset showing close up of third-fifth teeth of mandible) **G–J** labrum **G** exterior view of labrum **H** interior view of labrum **I** teeth on labrum (left margin from H) **J** teeth on labrum (right margin from H) **K** mandibular palp **L** close-up on superior side showing serrulated setae on mandibular palp **M–P** penis **M** overview of whole penis on side view and inset showing smooth dorsal side (dash line) on the base of the penis without basi-dorsal point **N** apex of penis carrying setae **O** penis with annulation **P** base of penis. Abbreviations: ul, upper lobe of maxilla; ll, lower lobe of maxilla. Scale bars in µm.

Maxilla bi-lobate; both lobes covered with serrulated setae (Fig. 8A, B, C). Maxillule with V-shaped notch; two large spines and five smaller spines before notch; six pairs of long slender spines and following seven smaller spines closed to inferior angle after notch (Fig. 8D); cutting edge after notch carrying a cluster of serrulated setae (Fig. 8E). Mandible with five teeth; the first teeth largest; the second and the third teeth bidentate; the fourth teeth serrated; the fifth teeth only single in the middle of lower margin surrounded by small and slender spines; lower margin narrow with a pack of 12 spines (irregular length); no setae under inferior angle (Fig. 8F). Labrum with V-shaped notch; four teeth with densely packed simple setae on each side of cutting margin (Fig. 8H–J). Mandibular palp rectangular carrying densely packed serrulated setae on superior margin (Fig. 8K–L). Penis long and annulated without basi-dorsal point (Fig. 8M inset); a few simple setae scattered randomly along whole length; at the tip of penis carrying two clusters of simple and long setae (Fig. 8M–P).

######### Habitat.

The specimens were collected only during the lowest tide (March) of the year at the lowest tide littoral zone, the same habitat as *Neonrosellavitiata*.

######### Distribution.

Western Pacific from Taiwan (type locality) and Philippines (Chan and Cheang 2016) and Andaman Sea of eastern Indian Ocean (Phang-Nga Province, southern Thailand).

####### Key to the western Pacific and the Andaman Sea of eastern Indian Ocean species of subfamily Newmanellinae

**Table:** 

1	Low-conic shell plate on calcareous base with four parietes; two layers of parietal tubes (inner laminar with radiating large tubes and outer laminar with three horizontal tubes) (Fig. 1B, C); cirrus IV without triangular spines (Fig. 3D); mandible with five teeth (Fig. 4F); penis with basi-dorsal point (Fig. 4O)	*** Neonrosella vitiata ***
–	Low-conic shell plate on calcareous base with four parietes; multiple layers of parietal tubes; cirrus IV with triangular spines (see Fig. 7D and Chan and Cheang 2016: fig. 5G); mandible with five teeth; penis without basi-dorsal point (Fig. 8)	**2**
2	External shell plate white and longitudinal fold from apex to base without colour spots; tergum with narrow spur (see Chan and Cheang 2016: fig. 2); the third teeth of mandible bidentate, the fourth teeth bidentate with cutting edge serrated and small teeth, and the fifth teeth close to the fourth (see Chan and Cheang 2016: fig. 7G, H); five teeth on each side of labrum (see Chan and Cheang 2016: fig. 8E)	*** Newmanella radiata ***
–	External shell plate green; scutal margin of tergum serrated (Fig. 5I); the third teeth of mandible bidentate, four serrated and small teeth close to base of the fourth teeth, the fifth teeth in the middle of pectin (Fig. 8F); four teeth on each side of labrum (Fig. 8H)	*** Newmanella spinosus ***

## Discussion

The present study represents the first discovery of *Neonrosellavitiata*, sharing overlapping habitat with *Newmanellaspinosus* in the Andaman Sea, eastern Indian Ocean. We previously reported a list of new record acorn barnacles in Thailand (the Gulf of Thailand and the Andaman Sea) and *New.spinosus* was also observed in the low-tide intertidal zone at Na-Tai District, Phang-Nga Province, southern Thailand (Pochai et al. 2017). The collection of new batches of specimens further down the rockweed at this region uncovered the presence of two newmanellin species (clearly recognized by their low conical shell plate with four parietes): one with white-background shell plates carrying decorations of dark orange spots and one with green shell plates. The white newmanellin species were thought to be *Newmanellaradiata* redescribed in Chan and Cheang (2016). However, based on the shell morphology characters (white shell plate with radiating orange stripes and two-layered and unequal-sized parietal tubes, tergum with broad spur and longer basal margin carrying extensive lateral depressor crests than that of *New.radiata*), this provides a possible clue for the occurrence of *Neonrosella*. By observation under the conical shell plate, *New.spinosus* is easily distinguished from *Neonrosella* in that they possess multiple layers (three or more) of parietal tubes in honeycombed pattern. Based on examination of arthropodal characters by scanning electron microscopy, *Neo.vitiata* carried different morphologies of cirri I–VI in the presence and absence of triangular spines on greater and/or lesser curvature of the anterior and/or posterior rami. Additionally, *Neo.vitiata* and *New.spinosus* exhibited unequal rami in cirri I and III but equal in others, as described in Table 1. Unique characters among several body parts were found in the mandible and labrum. In the mandible of *Neo.vitiata*, the third and fourth teeth are tridentate and quadridentate while both teeth are bidentate in *New.radiata*. There are three teeth on each V-shaped cutting edge of *Neo.vitiata* but five in *New.radiata*. The obvious difference between *Neonrosella* and *Newmanella* is found in their intromittent organ or penis, in that *Neo.vitiata* carries basi-dorsal point on the base of penis while both *New.spinosus* and *New.radiata* have smooth dorsal surface of penis base.

**Table 1.** Summary of shell plate morphology and anatomical characters used to diagnose *Neonrosellavitiata* from two morphologically related *Newmanella* species *New.radiata* and *New.spinosus*. Diagnostic characters to distinguish these three species are marked in bold.

**Characters**	***Neonrosellavitiata* (Darwin, 1854)**	***Newmanellaradiata* (Bruguière, 1789)** As redescribed in Chan and Cheang (2016)	***Newmanellaspinosus* Chan & Cheang, 2016** As redescribed in Chan and Cheang (2016) and the present study
Shell plates	Low conical; white with irregular longitudinal dark orange/brownish stripes	Low conical; white with radiating lines	Low conical; **green** with radiating lines
Parietal tube	Two layers: inner laminar with larger parietal tubes; outer laminar with three smaller parietal tubes between large parietal tubes from inner laminar	Two layers with irregular size of holes	**Three layers**
Tergum	Broad spur with rounded tip; ten lateral depressor crests on long basal margin; scutal margin without serrated teeth	**Narrow spur**; 4–5 lateral depressor crests on basal margin; scutal margin without serrated teeth	Broad spur with cutting edge; 9–10 Lateral depressor crests on basal margin; scutal margin **with serrated teeth**
Scutum	Triangular; height and width equal; deep and numerous lateral scutal depressor crests	Triangular; height and width equal; deep and numerous lateral scutal depressor crests	Triangular; height **longer than width** by 1.5 times; deep and numerous lateral scutal depressor crests
Cirrus I	1. Unequal rami; anterior ramus longer than posterior ramus 2.5 fold	1. Unequal rami; anterior ramus longer than posterior ramus 2/3 fold	1. Unequal rami; anterior ramus longer than posterior ramus
	2. Posterior ramus normal/**not protuberant**	2. Posterior ramus protuberant	2. Posterior ramus protuberant
Cirrus II	1. Equal rami	1. **Unequal rami**; posterior ramus longer 1.5 fold than anterior ramus	1. Equal rami
	2. Greater/lesser curvature of both rami without triangular spines	2. Greater/lesser curvature of both rami without triangular spines	2. Lesser curvature of both rami **with spines**
Cirrus III	1. Unequal rami; both antenniform	1. Unequal rami; only **posterior ramus antenniform**	1. **Semi-equal rami**; both antenniform
	2. Lesser curvature of anterior (only 7-segmented) and posterior rami (only 4-segmented) with triangular spines	2. Lesser curvature of anterior (entire) and posterior rami (not 3 distal segments) with triangular spines	2. Lesser curvature of anterior and posterior with triangular spines
	3. Greater curvature of anterior ramus without spines	3. Greater curvature of anterior ramus without spines	3. Greater curvature of anterior ramus with spines
Cirrus IV–VI	Basis with triangular spines	Basis with triangular spines	Basis **without triangular spines**
Cirrus IV	**No triangular spines** at greater curvature of anterior ramus	Triangular spines at greater curvature of anterior ramus	Triangular spines at greater curvature of anterior ramus
Mandible	Five teeth: 1^st^(1)+2^nd^(2)+ **3^rd^(3)**+4^th^(3-4/serrated) +5^th^(1)+7 small setae+3 larger setae at lower margin	Five teeth:1^st^(1)+2^nd^(2)+3^rd^(2)+4^th^(2/serrated)+5^th^(1)+16 setae at lower margin	Five teeth: 1^st^(1)+2^nd^(2)+3^rd^(2)+4^th^ (1/serrated)+5^th^(1)+12 setae at lower margin
Labrum	Three teeth on each side of cutting margin	Five teeth on each side of cutting margin	Two large teeth right side and five teeth on left side (in this study – 4 large teeth on right and left sides)
Penis	Penis long and annulated **with basi-dorsal point** and at the tip of penis carrying two clusters of simple and long setae	Penis long and annulated **without basi-dorsal point**	Penis long and annulated **without basi-dorsal point**

*Neo.vitiata* in this study exhibited some similarities in shell plate morphology to *Tetraclitavitiata* Darwin, 1854 found in Philippines and Indo-west Pacific water, as described in Rosell (1972) as following: i) white conical shell plate ii) a few layers of irregular parietal tubes iii) long basal margin of tergum with several lateral depressor crests. However, our redescription of *Neo.vitiata* here report more distinct feature in following terms: colouration of external and internal shell plate with dark orange spots/lines and other arthropodal characters, including less number of cirral segments, the presence of serrulated and bipinnate setal types, the presence of triangular spines on both anterior and posterior ramus in cirri III, cirri IV–VI with three pairs of unequal setae, and in particular penis carrying hair tuft-like in group of two with basi-dorsal point.

Across all regions we examined in both the Gulf of Thailand and the Andaman Sea, *Neo.vitiata* was found only at the rocky shore-rockweed interface of the intertidal zone during the lowest tides, and the only one site for sample collection is Na-Tai, Phang-Nga. However, further investigations of more sampling areas at deeper depths of the intertidal zone are required and they may reveal a subtidal distribution of this species. In addition, the presence of *Neo.vitiata* in eastern Indian Ocean provides a possible scenario that before sea levels fluctuated by glaciation during the Pleistocene (e.g., Voris 2000), *Neo.vitiata* was already distributed across the Pacific Ocean towards the Indian Ocean.

## Supplementary Material

XML Treatment for Neonrosella

XML Treatment for Neonrosella vitiata

XML Treatment for Newmanella

XML Treatment for Newmanella spinosus
